# Human Intraocular Filariasis Caused by *Dirofilaria* sp. Nematode, Brazil

**DOI:** 10.3201/eid1705.100916

**Published:** 2011-05

**Authors:** Domenico Otranto, Daniel G. Diniz, Filipe Dantas-Torres, Maurizio Casiraghi, Izabela N.F. de Almeida, Luciana N.F. de Almeida, Jeannie Nascimento dos Santos, Adriano Penha Furtado, Edmundo F. de Almeida Sobrinho, Odile Bain

**Affiliations:** Author affiliations: Università degli Studi di Bari, Bari, Italy (D. Otranto, F. Dantas-Torres);; Universidade Federal do Pará, Pará, Brazil (D.G. Diniz, I.N.F. de Almeida, J. Nascimento dos Santos, A. Penha Furtado, E.F. de Almeida Sobrinho);; Università degli Studi di Milano-Bicocca, Milan, Italy (M. Casiraghi);; Universidade Federal de Minas Gerais, Belo Horizonte, Brazil (L.N.F. de Almeida);; Muséum National d’Histoire Naturelle, Paris, France (O. Bain)

**Keywords:** Dirofilaria sp., nematode, intraocular filariasis, zoonosis, human, eye, parasites, Brazil, dispatch

## Abstract

A case of human intraocular dirofilariasis is reported from northern Brazil. The nematode was morphologically and phylogenetically related to *Dirofilaria immitis* but distinct from reference sequences, including those of *D*. *immitis* infesting dogs in the same area. A zoonotic *Dirofilaria* species infesting wild mammals in Brazil and its implications are discussed.

Zoonotic filariases are caused by nematodes of the superfamily Filarioidea and are transmitted by blood-feeding arthropods. Within this taxonomic group, nematodes of the genus *Dirofilaria* are among the most common agents infecting humans (*1**–**5*). The 2 main species of zoonotic concern are *Dirofilaria immitis*, which causes canine cardiopulmonary disease, and *D*. *repens*, which is usually found in subcutaneous tissues. In addition, zoonotic subcutaneous infections with *D*. *tenuis* in raccoons and *D*. *ursi* in bears have been reported less frequently in North America (*3*). *Dirofilaria* spp. infections in humans have been detected mostly in subcutaneous tissue and lungs (*3*), and 1 intraocular case of infection with *D*. *repens* was reported from Russia (*1*). *D*. *immitis* and *D*. *repens* are the main causes of human dirofilariasis in the Americas (*4**,**6*) and Old World (*2**,**5*), respectively.

Morphologic identification of *Dirofilaria* spp. is based on the body cuticle, which is smooth in *D*. *immitis* (subgenus *Dirofilaria*) and has external longitudinal ridges in *D*. *repens* and other species of the subgenus *Nochtiella*. However, in many reported cases of zoonotic dirofilariasis, specific identification was not adequately addressed (*5*). Twenty-eight cases of human dirofilariasis in the Old World attributed to *D*. *immitis* have been reviewed recently and attributed to *D*. *repens* (*5*). On the basis of this information, the suggestion was made that *D*. *immitis* populations have different infective capabilities for humans in the Old and New Worlds (*5*). However, this hypothesis was not supported by recent genetic comparisons of specimens from the Old World (Italy and Japan) and New World (United States and Cuba) (*7*; M. Casiraghi, unpub. data).

We report a case of human intraocular dirofilariasis in which a live male *Dirofilaria* sp. worm was recovered from the anterior chamber of a patient’s eye. Morphologic and molecular studies suggested that this zoonotic case of dirofilariasis was caused by a *Dirofilaria* sp. closely related to *D*. *immitis*.

## The Study

**Figure 1:**
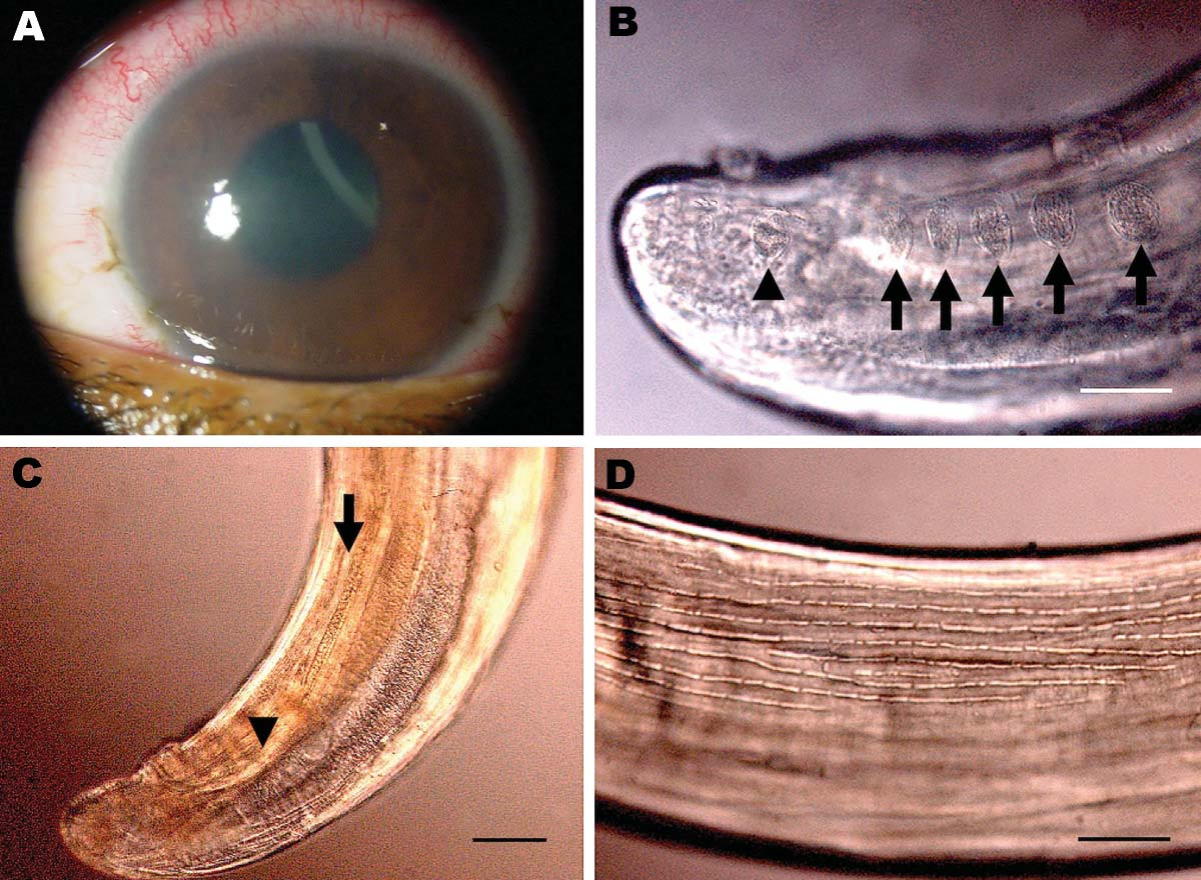
Corneal edema and episcleral hyperemia in the left eye of a 16-year-old boy from Brazil and a free-swimming filarid in the anterior chamber. A) Macroscopic view. B) Five pairs of ovoid pre-cloacal papillae (arrows) and 1 postcloacal caudal papillae (arrowhead). Scale bar = 50 µm. C) Small (arrowhead) and large (arrow) spicules. Scale bar = 40 µm. D) Longitudinal ridges of the area rugosa. Scale bar = 50 µm.

On September 16, 2008, a 16-year-old boy came to the Clínica de Olhos do Pará in Pará, Brazil, with low visual acuity (0.54 m), an intraocular pressure of 44 mm Hg, and pain in the left eye. Ophthalmologic examination showed a nematode (Video) in the anterior chamber of the left eye. The patient reported no travel history in recent months preceding the onset of symptoms. Corneal edema (Figure 1, panel A) and episcleral hyperemia in the left eye were observed, and surgery for removal of the nematode was recommended. After peribulbar anesthesia, the eye was clipped and the cornea was incised with a crescent Beaver corneal knife, and the nematode was extracted with forceps and Fukasacu hook (Video). A live filarial nematode was removed from the anterior eye chamber. The patient recovered without complications after surgery.

**Video:**
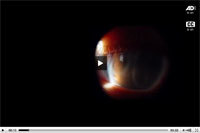
Surgical removal of a *Dirofilaria* sp. nematode from the left eye of a 16-year-old boy, Brazil. VA, visual acuity; CF, count fingers; IOP, intraocular pressure. A portion of the material in this video was previously published in the journal Parasites and Vectors (http://www.parasitesandvectors.com/content/pdf/1756-3305-4-41.pdf).

Direct Link: http://streaming.cdc.gov/vod.php?id=b35b57a52f8e7b3e41b59328391c90b020110426172643263

The parasite (deposited at the Muséum National d’Histoire Naturelle, Parasitologie Comparée, Paris, France; accession no. 143 YU) was a male filarial nematode (length 106 mm, width 400 µm). It had large caudal alae, several pairs of pedunculated papillae (Figure 1, panel B), unequal spicules, a short and thick tail, and an esophagus with a wider glandular region.

Narrow hypodermal lateral chords and the 2 lateral internal cuticular crests are typical for *Dirofilaria* spp (*8*). The specimen was not *D*. *repens* or a *Nochtiella* species because of the smooth body cuticle. The left spicule (length 378 µm), with a handle and lamina of equal length, was similar to that of *D*. *immitis* (Figure 1, panel C). Other measurements were similar to those of *D*. *immitis* (esophagus length 1,200 µm; right spicule length 170 µm; tail length 110 µm; width at the anus 110 µm) (*8*).

To confirm morphologic identification, a DNA barcoding approach was used (*7*). A small piece (≈3 mm) of the nematode was used for DNA extraction and amplification of cytochrome c oxidase subunit 1 (*cox1*) and 12S rDNA gene fragments as described (*9*). Sequences were deposited in the European Molecular Biology Laboratory Nucleotide Sequence Database (accessions nos. HQ540423 and HQ540424). In accordance with morphologic identification, BLAST (http://blast.ncbi.nlm.nih.gov) analysis of both genes showed overall highest nucleotide similarity with those of *D*. *immitis* available in GenBank (12S rDNA, accession nos. AM779770 and AM779771; *cox1*, accession nos. AM749226 and AM749229).

**Figure 2:**
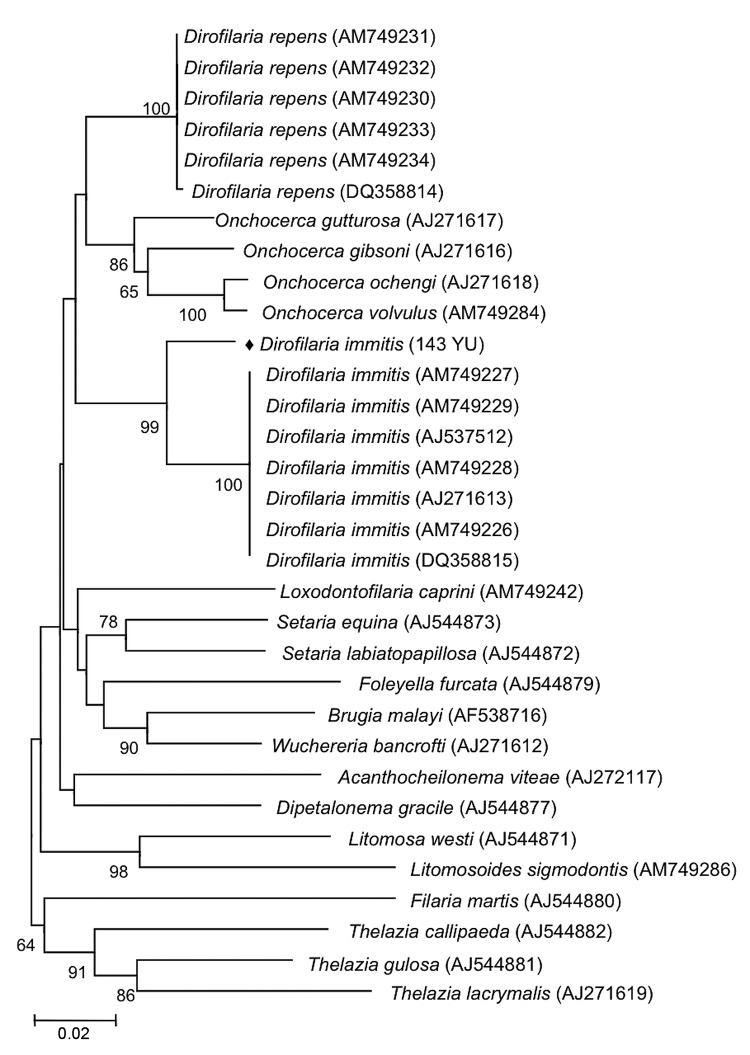
Phylogeny of filarial nematodes based on cytochrome c oxidase subunit 1 (*cox1*) gene sequences. *Thelazia* spp. species were used as outgroup. Bootstrap confidence values (100 replicates) are shown at the nodes only for values >50%. Solid diamond indicates nematode isolated in this study. Numbers in parentheses are GenBank accession numbers. Scale bar indicates nucleotide substitutions per site.

Phylogenetic analysis using *cox1* sequences by MEGA 4.0 (www.megasoftware.net), a neighbor-joining algorithm, and Kimura 2-parameter correction confirmed that 143 YU clustered with *D*. *immitis* from different countries, including Australia (GenBank accession no. DQ358815) and Italy (other sequences in Figure 2). The *cox1* sequence of *D*. *immitis* from a dog in Pará, Brazil, was identical with those of the same species in GenBank. Topology of 12S rDNA sequences was identical with that of *cox*1 sequences. However, comparison of nematode sequences with those in GenBank showed large differences in nucleotide similarities (5% and 6% for 12S rDNA and *cox1*, respectively).

Morphologic comparison with male worms isolated from dogs (1 from People’s Republic of China and 1 from Japan, Muséum National d’Histoire Naturelle accession nos. 63 SE and 169 YU) showed that the area rugosa (ventral ornamentation of the posterior region) was similar among all specimens examined and was composed of ≈8 longitudinal crests, each containing aligned segments (length 15–30 µm) extending 1–8 mm from the tail tip (Figure 1, panel D). Arrangement and number of caudal papillae were also similar (*10*). The preesophageal cuticular ring was present in *D*. *immitis* reference specimens but was not present in the specimen from Brazil. In addition, the deirids were more anterior in the worm from Brazil than in reference specimens. This finding might be caused by less growth of the nematode found in the patient from Brazil.

Similar morphologic features (small size and anterior deirids) have been reported in a *D*. *immitis* male worm from a dog in Cayenne, French Guiana (*11*). However, a *D*. *spectans* worm from an otter in Brazil had a similar body size and location of deirids as the worm 143 YU but a different pattern of juxtacloacal papillae.

## Conclusions

In Brazil, human pulmonary dirofilariasis caused by *D*. *immitis* has been reported sporadically (*12*), mainly in southeastern Brazil, where the prevalence of heartworm in dogs ranges from 2.2% to 52.5% (*13*). The patient in this study came from a region in Brazil where canine dirofilariasis caused by *D*. *immitis* is endemic and in which the prevalence of microfilaremic dogs is <32.5% (*14*). Morphologic features and overall high identity of 12S rDNA and *cox1* gene sequences compared with the reference DNA sequences confirmed that the worm recovered was similar to *D*. *immitis*. However, nucleotide similarity differences in comparison with other sequences of *D*. *immitis* available in GenBank (including 1 from Brazil) were 5% and 6% for 12S rDNA and *cox1* genes, respectively, which are higher than the range of intraspecific variation (<0.8%) reported for *D*. *immitis* originating from the United States, Italy, and Japan (*7*). Such variation (>5%) has not been reported for a *Dirofilaria* species. Therefore, existence of a closely related species (e.g., *D*. *spectans* from otters, but also reported from a human in Brazil) (*15*) should be considered. Further investigations of filarial nematodes from dogs and wild mammals in Brazil are required to elucidate the identity of this *Dirofilaria* species and its primary hosts and vectors.
